# Development of High-Performance Soft Robotic Fish by Numerical Coupling Analysis

**DOI:** 10.1155/2018/5697408

**Published:** 2018-11-27

**Authors:** Wenjing Zhao, Aiguo Ming, Makoto Shimojo

**Affiliations:** ^1^College of Mechanical Engineering, Zhejiang University of Technology, Hangzhou 310014, China; ^2^Department of Mechanical Engineering and Intelligent Systems, The University of Electro-Communications, Tokyo 182-8585, Japan

## Abstract

To design a soft robotic fish with high performance by a biomimetic method, we are developing a soft robotic fish using piezoelectric fiber composite (PFC) as a flexible actuator. Compared with the conventional rigid robotic fish, the design and control of a soft robotic fish are difficult due to large deformation of flexible structure and complicated coupling dynamics with fluid. That is why the design and control method of soft robotic fish have not been established and they motivate us to make a further study by considering the interaction between flexible structure and surrounding fluid. In this paper, acoustic fluid-structural coupling analysis is applied to consider the fluid effect and predict the dynamic responses of soft robotic fish in the fluid. Basic governing equations of soft robotic fish in the fluid are firstly described. The numerical coupling analysis is then carried out based on different structural parameters of soft robotic fish. Through the numerical analysis, a new soft robotic fish is finally designed, and experimental evaluation is performed. It is confirmed that the larger swimming velocity and better fish-like swimming performance are obtained from the new soft robotic fish. The new soft robotic fish is developed successfully for high performance.

## 1. Introduction

With the development of interdisciplinary sciences, including the science of electronic information and biological technology, biomimetic robots have made a lot of irreplaceable contributions in human life [1, 2]. Many researchers have been dedicated to the fields of biomimetic robots, especially on the development of a biomimetic robotic fish by mimicking the real fish [3–7]. The biomimetic robotic fish, expressed as high efficiency, good mobility, and small disturbance, will be widely available for the rescue, exploration and observation of the seabed, and other special tasks.

In order to fulfil some special work in the complicated or unknown environment accurately and reliably, the biomimetic robotic fish should be designed as soft as the real fishes, and the actuation strategy using a soft structure is needed due to the obvious advantages of smooth propulsion, high safety, and high mobility. It has been proven difficult to reproduce the fish-like smooth propulsion by using conventional rigid mechanisms, causing a relatively complex and unreliable propulsion structure with low efficiency and mobility [8, 9]. A soft robotic fish combining soft behaviors of real fishes with robotics is thus gradually turning up [6, 7, 10–13]. As a young field, the relative theories and technologies have not yet been defined in a general method and activities of robot development are still exploring the new ways [8, 13]. Therefore, it motivates us to challenge the design and control of the soft robotic fish by biomimetic approach for the development of a high-performance soft robot. Many soft robots use flexible actuators with smart materials for smooth propulsion, such as shape memory alloy (SMA), electrostatic film, PZT film, ionic polymer metal composite (IPMC), or PFC. Based on the propulsion mechanism using flexible actuators, the soft robotic fish can obtain relatively better propulsion performances similar to those of real fishes.

When designing a soft robotic fish, it must consider a surrounding fluid. The fluid increases the system mass, stiffness, and damping and changes the dynamic characteristics of the soft robotic fish. The fluid-structural coupling analysis considering the interaction between the flexible structure and the surrounding fluid is performed in the design of dynamic behaviors of the soft robotic fish. Most of fluid-structural coupling problems involve a fluid without significant flow, and the main concern in the fluid is pressure wave propagation. The acoustic fluid-structure coupled method considering the sound pressure can thus be utilized to solve these coupling problems and predict their dynamic responses. According to the coupling analysis using finite element method (FEM), Ai and Sun [14] studied the vibration response of underwater cylindrical shells, Sung and Nefske [15] analyzed dynamic responses of an automobile compartment, and Djojodihardjo and Safari [16] achieved the characteristics of spacecraft structures, etc. These researches provided a basis of flow formulation and boundary conditions. However, the coupling problem is very difficult to be solved, and the design and control of the soft robotic fish have not been established due to large deformation and complicated coupling dynamics with fluid. It is necessary to investigate the design and control problems of the soft robotic fish by analytical simulation with a coupling effect.

In the present research, the fish's body and/or caudal fin (BCF) propulsion is focused. PFC can be constructed to a simple structure with high energy conversion efficiency and large displacement response and is utilized as a soft actuator to develop a BCF-propulsion prototype. In the paper, the acoustic fluid-structural coupling using FEM is applied to predict dynamic responses of the soft robotic fish in the fluid. Through the coupling analysis, a new soft robot structure is proposed, and a well-established experiment is performed to evaluate dynamic behaviors with the coupling method. From the results, it is confirmed that the new soft robotic fish with larger velocity and better fish-like swimming performance is achieved by the established coupling method. The new high-performance soft robotic fish is developed successfully.

## 2. Materials and Methods

### 2.1. Piezoelectric Fiber Composite

**Figure 1:**
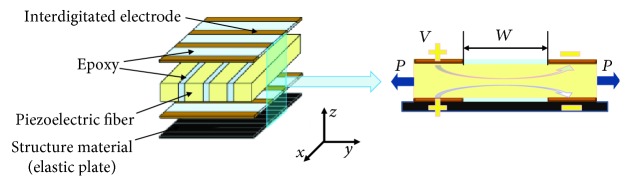
Structure and driving model of MFC.

In this study, macrofiber composite (MFC) [17], one of the typical PFC, was adopted as a soft actuator.  Figure 1 shows the structure model of MFC. The piezoceramic fiber was embedded in epoxy to make a rectangular plate. The plate was sandwiched by two pieces of polyimide film on which the interdigitated electrodes were placed. When a voltage from −500 V to +1500 V was applied, the strain and stress would be generated, and an equivalent driving load could be finally obtained by (1) for expansion and contraction in the direction of the fibers [18]:

(1) $$\begin{array}{c} \left\{\begin{array}{c} P_{x}\\ P_{y} \end{array}\right\}=\frac{1}{1-v_{xy}v_{yx}}\left[\begin{array}{cc} E_{x} & E_{x}v_{yx}\\ E_{y}v_{xy} & E_{y} \end{array}\right]\left\{\begin{array}{c} d_{33}\\ d_{31} \end{array}\right\}\frac{V}{W}, \end{array}$$

where *E*_*x*_ and *E*_*y*_ are the tensile modulus in the X and Y directions, respectively, *v*_*xy*_ and *v*_*yz*_ are Poisson's ratio, *d*_33_ and *d*_31_ are the piezoelectric constants, *V* is the voltage, *W* is the distance between the electrodes, and *P* is the driving load.

When MFC combined with a thin elastic plate, the bending deformation generated due to resonance. The characteristics of bending deformation were utilized to design the soft robotic fish with BCF propulsion. The carbon-fiber-reinforced polymer (CFRP) was adopted as the thin elastic plate in the research.

### 2.2. Design Method and Numerical Coupling System of Soft Robotic Fish

**Figure 2:**
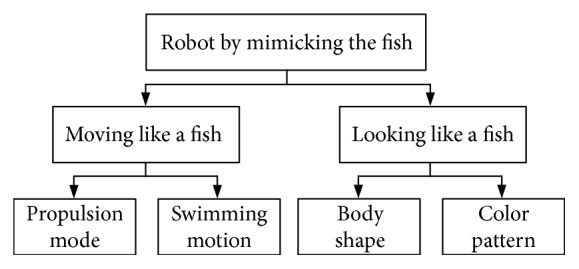
Decomposition of functions for a soft robot mimicking the fish.

The biomimetic approach has been used to design the soft robotic fish in the unknown or complicated environments. Through mimicking the real fish, the concerned functions of the soft robot can be classified into two types: moving like a fish and looking like a fish, described in Figure 2. The type of moving like a fish focuses on the propulsion modes and motions similar to those of real fishes. The swimming performances such as velocity and swimming number are adopted to evaluate the propulsion performance of the soft robot. For the type of looking like a fish, it concerns a fish species by matching its body shape and color pattern [19]. In the study, the function of moving like a fish was mainly focused.

**Figure 3:**
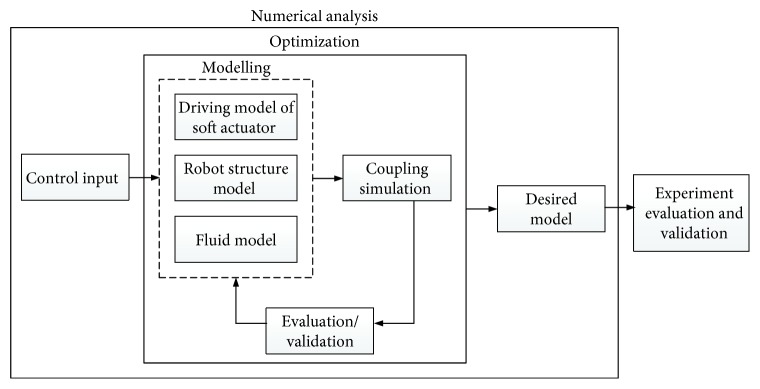
Design procedure of the soft robotic fish.

In the research, the main research purpose is to develop the high-performance soft robotic fish by a biomimetic method. The design method based on numerical analysis is presented. It takes into account the interaction between the flexible structure and the surrounding fluid.  Figure 3 shows the design procedure of the soft robotic fish. The desired robot model is firstly proposed through the coupling analysis. The manufacture and experiment evaluation are then carried out for validation. In the research, the fish with subcarangiform type such as trout fish is focused. The propulsion motion is dominated by the inertial force of the fluid [20]. The driving model based on an MFC soft actuator is described, in which the control input is considered. Through the driving model, the modelling of the soft robotic fish including the surrounding fluid is presented, and the corresponding material properties are defined. Based on the modelling, the numerical coupling simulation is performed to establish the fish-like propulsion motion, and then the robot structure is optimized by the established coupling simulation for improvement until the achievement of the desired model. When the desired model is achieved, the experiment evaluation will be done by using the actual prototype.

### 2.3. Approach of Acoustic Fluid-Structural Coupling

In this paper, the FEM approach was used to deal with the acoustic coupling problems through ANSYS software. Acoustic elements, four degrees of freedom (DOF): three for optional displacement and one for pressure, accomplished the required fluid-structural coupling. A consistent matrix coupling was set up between structural and fluid elements in which strongly coupled physics caused no convergence problems.

In the fluid-structural coupling, a flexible robot structure was in contact with an enclosed fluid. The differential motion equation of a continuum body in finite element formulation was used to model the flexible robot structural domain. The fluid domain presented by the acoustic wave equation met the basic conditions: inviscid, compressible, no mean flow, no heat transfer process, and relatively small pressure. The amplitude in the fluid domain was relatively small for small variation in fluid density. The mean density and pressure were uniform throughout the fluid domain. The interaction between the flexible robot structure and the surrounding fluid at the interface caused the fluid pressure to exert a force applied to the flexible robot structure, and the structure motion produced an effective load acting on the fluid. The coupling boundary conditions at the interface met the continuity of displacement and fluid pressure between two domains. Through the coupling boundary condition and algorism, the soft robotic fish's coupling problem could be described by an unsymmetrical system in a finite element matrix equation shown as (2). Both structural and fluid loads were transferred at the interface. The nodes on the interface had both displacement and pressure DOF. If the body force *F*_*S*_ is zero, (2) will become the equation of coupled modal analysis:

(2) $$\begin{array}{c} \left[\begin{array}{cc} M_{S} & 0\\ \rho R^{T} & M_{F} \end{array}\right]\left\{\begin{array}{c} U\\ P \end{array}\right\}+\left[\begin{array}{cc} K_{S} & -R\\ 0 & K_{F} \end{array}\right]\left\{\begin{array}{c} U\\ P \end{array}\right\}=\left\{\begin{array}{c} F_{S}\\ F_{F} \end{array}\right\}, \end{array}$$

where *M* and *K* are the structural element mass matrix and stiffness matrix, respectively, *F* is the body force, *U* is the displacement, *P* is the fluid pressure, *ρ* is the fluid density, and subscript *S* and *F* are expressed as the structural domain and fluid domain, respectively. *R*, the fluid-structure coupled matrix, represents the effective surface area associated with each node on the fluid-structure interface and considers the direction of the normal vector defined for each pair of coincident fluid and structure element faces that comprises the interface surface.

## 3. Structure Design Based on Coupling Analysis

### 3.1. Structure of Soft Robotic Fish

**Figure 4:**
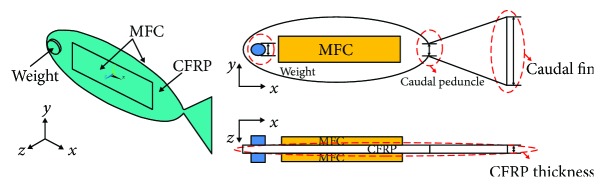
Structure of the soft robotic fish.

Figure 4 shows the structure of the soft robotic fish. The robot body was made by a CFRP plate and two MFC plates. Two MFC plates using M-8528-P1 type sandwiched the CFRP plate as an actuator structure. The weight made by steel was placed on the robot head to increase the displacement of the tail end. The height of the robot body was varied toward the tail end, and it was smallest at the place of the caudal peduncle. The material properties of the soft robotic fish are shown in Table 1.

### 3.2. Design Scheme of Soft Robotic Fish

The real fishes move forward by making transformation waves generated by a meandering motion. The transformation is obtained by the bending moment generated from the muscles along the body axis of real fishes. In the meandering motion of the real fish, the inertia force apparent increase in fluid mass becomes a propulsive force in the increased amplitude case. The net positive force can be obtained at the locomotion direction when the motion amplitude is increased toward the tail [20]. Moreover, in the propulsion of the trout fish [20], the undulation amplitude is limited anteriorly and increases only in the posterior half of the fish body; that is to say, there is increased amplitude from anterior to posterior of the fish body. Therefore, to design a soft robotic fish with large propulsive velocity, the motion amplitude needs to be increased toward the tail, and the maximum value should be located at the caudal fin end.

The main bending propulsion motion and the motion amplitude at the caudal fin end were focused in the design. To achieve the fish-like propulsion motion with large velocity, the structure design on larger amplitude at the tail end was carried out by the coupling analysis. The caudal fin shape, head weight, and body thickness of the soft robot played the important role in the propulsion [20, 21], and they were viewed as the key structural parameters in the design. The dashed lines in Figure 4 described the detailed locations of the design. The caudal peduncle height and caudal fin height were considered in the design of caudal fin shape. The CFRP plate as the main body of the actuation structure was important for large bending amplitude. Weight placed on the robot head was utilized to increase the amplitude of the tail end. These key parameters were allowed to vary within a certain range presented in Table 2. The caudal peduncle and caudal fin height were in the range of 1 mm~64 mm. The body thickness was ranged from 0.1 mm to 1 mm, and the radius of head weight was in the range of 1 mm~7 mm. These key parameters were arranged and combined at random to achieve the maximum displacement of the caudal fin end for improvement.

### 3.3. Fluid-Structural Coupling Analysis

**Figure 5:**
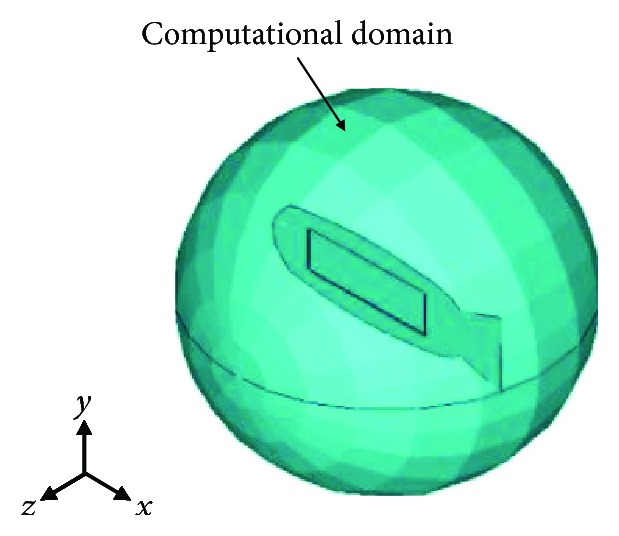
Computational domain of the soft robot fish in the fluid.

The coupled transient analysis was utilized to identify the dynamic behaviors of the soft robot in the fluid. The robot structural domain was described by three-dimensional (3-D) SOLID186 elements, and 3-D FLUID30 elements were used to model the fluid domain and coupled interface. As shown in Figure 5, a spherical surface was set as the boundary of the computational fluid domain, whose diameter was 220 mm. The soft robot was placed in the middle of the static fluid domain. Due to a high voltage of MFC, which could reach up to 2000 V, the Fluorinert Electronic Liquid FC3283 was adopted as insulating liquid, whose dielectric strength range was larger than 35 KV and density was 1820 kg/m^3^. It was a fully fluorinated liquid, and composition would not shift with time, which insured the fluid transport properties were stable.

**Figure 6:**
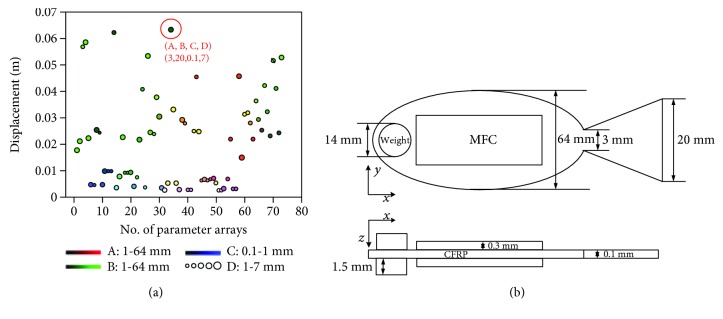
Model of the soft robot. (a) Displacement of caudal fin end based on different parameter arrays. (b) New model of the soft robot.

In the coupling analysis, the driving load of 3 Hz ranging from −500 V to +1500 V in sine waveform was applied. The simulation results on the displacement of the caudal fin end based on different parameter arrays are described in Figure 6(a). It could be known that the maximum displacement of the caudal fin end was about 62 mm. Based on this maximum value, the corresponding structural parameters could be identified, which were the caudal peduncle height of 3 mm, caudal fin height of 20 mm, body thickness of 0.1 mm, and radius of head weight of 7 mm. A new robot model was therefore designed and is shown in Figure 6(b).

The bending mode frequencies of the new robot model in the fluid are shown in Table 3, where the first three modes were presented. The first bending mode frequency was about 2.5 Hz, and its maximum deformation occurred at the tail end. The second bending mode similar to S-shape was obtained at about 9.5 Hz. If the frequency was close to 12 Hz, the third bending mode occurred.

## 4. Experiment Evaluation

**Figure 7:**
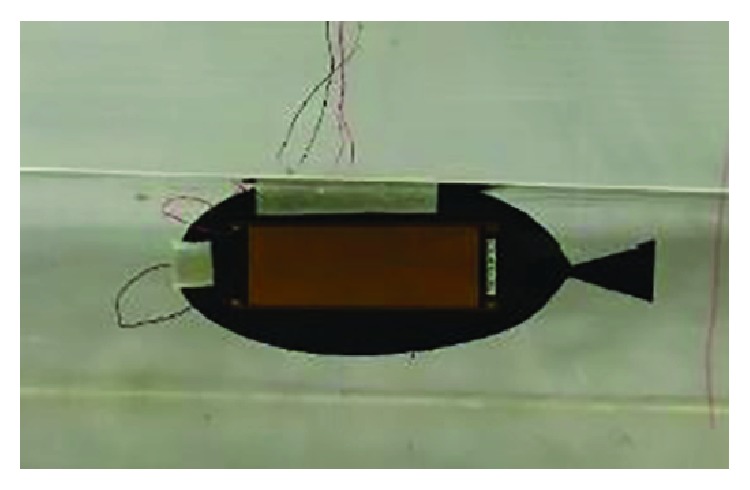
Prototype of the new soft robotic fish.

Figure 7 presents the prototype of the new soft robot. The low-density blowing agent as the float was placed on the robot head and top part to balance the robot weight in the fluid. The detailed specifications are shown in Table 4. The epoxy 3M-DP460 was used to make the MFC and CFRP plates bond together.

**Figure 8:**
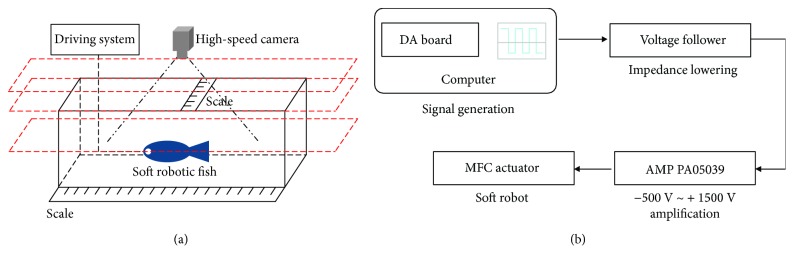
Experiment of robot prototype. (a) Experimental platform. (b) Driving system of the robot prototype.

Figure 8 presents the experiment platform and driving system of the prototype. A cubical fluid tank filled with Liquid FC-3283 and a high-speed camera (Digital Camera EX-F1 from Casio Company) were utilized in the experiment. The main signal generation was controlled by a computer, and basic signal waveforms such as sine wave were designed. The signal processing circuit used a voltage follower between a high-voltage amplifier (AMP PA05039) and a computer to reduce output impedance. The high-voltage amplifier was capable of delivering an output voltage of −500 V to +1500 V at an output current up to 50 mA DC. The high-voltage output was controlled by the amplifier whose input voltage was −2.5 V~7.5 V DC or peak AC corresponding to −500 V~+1500 V output. In the experiment, the input voltage was ranged from −500 V to +1500 V and the frequency was in the range of 1 Hz~20 Hz.

The bending mode frequencies of the prototype in the fluid are shown in Table 5. The main bending propulsion mode occurred at about 2 Hz, and the second and third bending modes generated at the frequency larger than 10 Hz. The simulation results were validated by the experiment.

**Figure 9:**
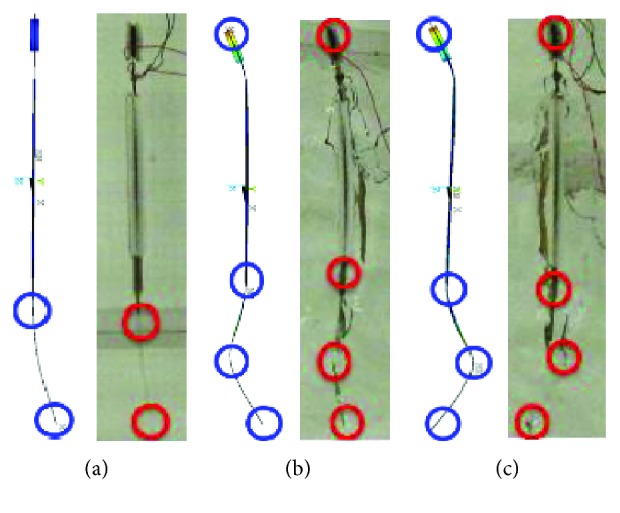
Bending propulsion modes of the new soft robot prototype in simulation and experiment. (a) First bending propulsion modes at 2 Hz. (b) Second bending propulsion modes at 10 Hz. (c) Third bending propulsion modes at 12 Hz.

The corresponding bending propulsion modes are shown in Figure 9 by a high-speed camera. There were very similar bending modes that occurred between the simulation and the experiment at the corresponding mode frequencies. When the frequency was about 2 Hz, the maximum displacement was obtained at the tail end. If the second and third bending modes happened, the maximum deformation generated at different parts of the robot body. The simulation results on bending propulsion modes about frequencies and mode shapes coincided with experimental results well, and fish-like bending propulsion motion was achieved.

**Figure 10:**
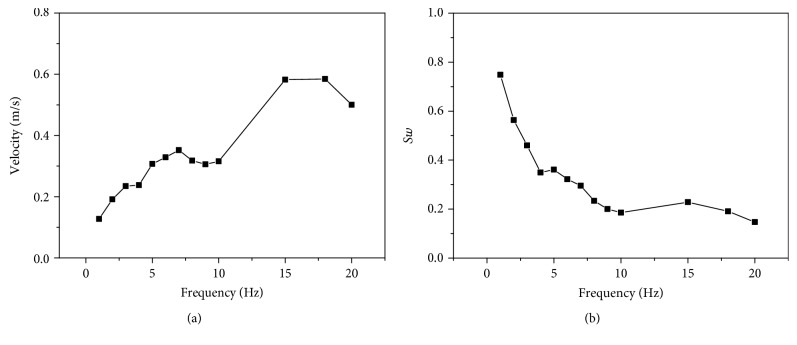
Swimming performance of the new soft robotic fish. (a) Swimming velocity at different frequencies. (b) Swimming number at different frequencies.

The new prototype also could realize the basic swimming motion such as the straight-ahead motion. The swimming velocity is described in Figure 10(a). At 15 Hz, the new prototype obtained the maximum velocity of about 0.6 m/s. In the biomimetics, the swimming number *S*_*w*_ of the fish was widely used to evaluate the swimming performances of the robotic fish [20]. *S*_*w*_, related to velocity *V*, frequency *f*, and body length *L*, could be expressed by

(3) $$\begin{array}{c} S_{w}=\frac{V}{fL}. \end{array}$$

*S*_*w*_ of the fish described the distance fish moved per tail beat. It was generally about 0.6 for high performances with good flexibility and mobility [20]. It meant that there was a representation of the premise of similarity law in the biomimetics. *S*_*w*_ was sufficient to describe the performance related to the swimming motion of the real fish by biomimetic approach [22].  Figure 10(b) presents the *S*_*w*_ of the new soft robot. It was decreasing with the increased frequency in the certain range. Maximum *S*_*w*_ was about 0.75 occurred at 1 Hz. At 2 Hz, *S*_*w*_ was close to 0.6, much closer to the value of real fishes. The new soft robotic fish had better fish-like propulsion performance near the main propulsion mode.

Table 6 presents the comparison of swimming performances among some conventional soft fish robots, considering the maximum velocity and *S*_*w*_. By comparison, the new soft robot had better fish-like propulsion performances on maximum swimming velocity by using speed (BL/s, body length per second) and *S*_*w*_. The maximum velocity of the new soft robot was improved by more than one order of magnitude. And *S*_*w*_ was enhanced by at least 3 times. It could be considered that the new soft robotic fish had higher propulsion performance similar to those of real fishes than the conventional soft robotic fish.

By comparison, the new soft robot had better fish-like propulsion performances on maximum swimming velocity by using speed (BL/s, body length per second) and *S_w_*. The maximum velocity of the new soft robot was improved by more than one order of magnitude. And *S*_*w*_ was enhanced by at least 3 times. It could be considered that the new soft robotic fish had higher propulsion performance similar to those of real fishes than the conventional soft robotic fish.

## 5. Turning Motion Control

By controlling the amplitude of input voltage on soft actuators, the soft robot could obtain the turning motion. The soft robot in [6] was taken as an example to describe in this part. The input voltage of 3 Hz ranging from −500 V to +1500 V in sine and square waveform was applied on this soft robot.

**Figure 11:**
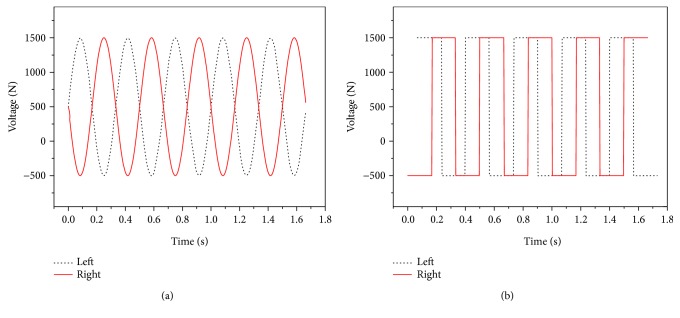
Waveforms on both MFC actuators at 3 Hz in straight-ahead motion. (a) Input voltages in sine waveform. (b) Input voltages in square waveform.

In the research, the soft robotic fish was composed of two soft actuators for propulsion. When the input voltage signals on both left and right MFC actuators had the same voltage amplitude and were distributed in opposite directions from each other, the straight-ahead motion was realized on the soft robotic fish.  Figure 11 describes the input voltages in sine and square waveform on both left and right MFC actuators for straight-ahead motion of the soft robotic fish. The input voltages on both actuators are all in the range of −500 V~+1500 V, and they were applied on the soft robotic fish in the opposite directions.

**Figure 12:**
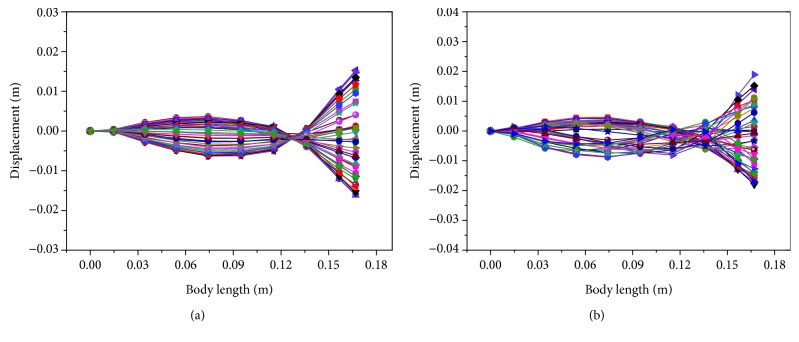
Propulsion modes of the soft robotic fish in one cycle at 3 Hz in straight-ahead motion. (a) Results in sine waveform. (b) Results in square waveform.

Based on these input voltage signals on both MFC actuators, the straight-ahead motion of the soft robotic fish was generated and the corresponding propulsion modes are presented in Figure 12. In Figure 12, the positive displacement described the displacement on the left side of the swing. Otherwise, it described the right-side displacement of the swing. The swing of the caudal fin in straight-ahead motion was in symmetrical distribution around the midline of the soft robotic fish in both sine and square waveform. Through characterizing this propulsion mode, the straight-ahead motion was realized. Besides, the swing displacement at the tail end in square waveform was larger. This was because the square waveform not only considered fundamental frequency component but also contained harmonics of fundamental frequency. It was possible that the soft robot had the faster swimming velocity by using the square waveform.

**Figure 13:**
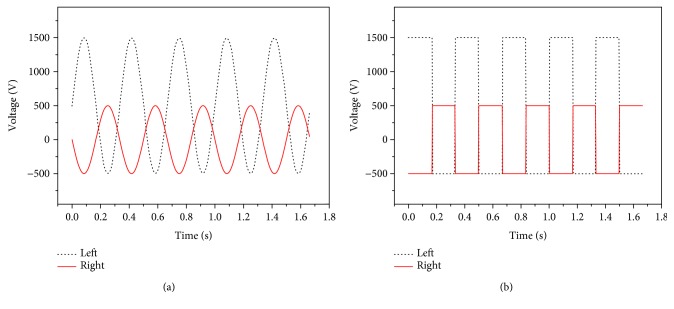
Waveforms on both MFC actuators at 3 Hz in the motion of turning right. (a) Input voltages in sine waveform. (b) Input voltages in square waveform.

In order to realize the turning motion of the soft robotic fish, the asymmetric signals were applied based on the conditions of the straight-ahead motion. Through controlling input voltage signals for bias voltage between two soft actuators, the turning motion could be obtained due to the asymmetrical output from actuators. When the voltage amplitude on the left actuator was larger, the motion of turning right was obtained. On the contrary, the motion of turning left occurred.  Figure 13 presents the input voltages on both actuators in the motion of turning right. The input voltage on the left actuator was in the range of −500 V~+1500 V, larger than the voltage ranged from −500 V to +500 V on the right actuator.

**Figure 14:**
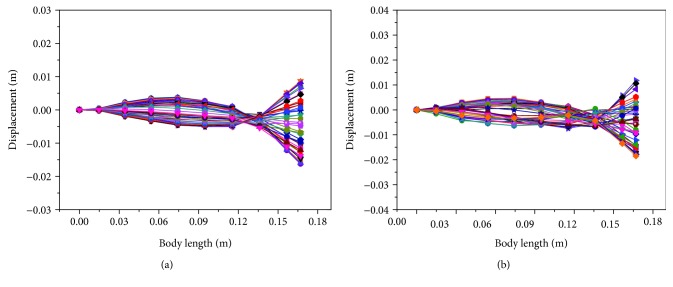
Propulsion modes of soft robotic fish in one cycle at 3 Hz in the motion of turning right. (a) Results in sine waveform. (b) Results in square waveform.

Based on these asymmetrical signals, the motion of turning right was achieved and corresponding propulsion modes are described in Figure 14. The swing of the caudal fin was asymmetrical around the midline of the soft robotic fish in both sine and square waveform. In Figure 14, the positive displacement described the displacement on the left side of the swing. Otherwise, it described the right-side displacement of the swing. The swing of the caudal fin mainly occurred on the right side of the soft robot, and the turning right motion thus generated. Besides, the swing displacement at the tail end in sine waveform was smaller than the results of square waveform due to the representation of only fundamental frequency in sine waveform.

**Figure 15:**
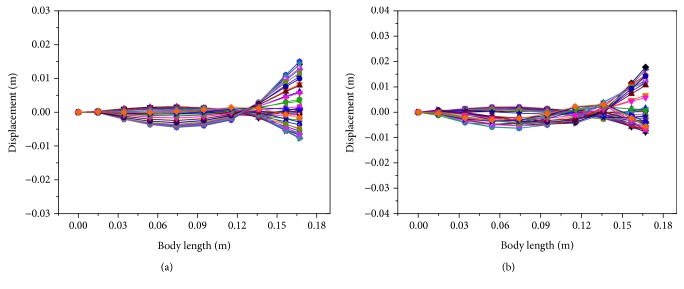
Propulsion modes of soft robotic fish in one cycle at 3 Hz in the motion of turning left. (a) Results in sine waveform. (b) Results in square waveform.

For the motion of turning left, the applied approach of input voltages was contrary to that of turning right motion. The voltage on the left actuator was in the range of −500 V~+500 V, and the voltage on the right actuator was ranged from −500 V to +1500 V. Based on these asymmetrical input voltages, the turning left motion was achieved and the corresponding propulsion modes are presented in Figure 15. The displacement on the left side of the swing was larger. It could also be found that the displacement at the tail end in sine waveform was smaller.

Based on the controllability of input signals, swimming motions such as turning motion could be achieved. To validate the turning motion control of the soft robot, the corresponding experiments on robot prototype in [10] were performed. The experimental platform on the measurement of the turning motion by a high-speed camera is described in Figure 8. The high-speed camera was placed on the top of the fluid tank. The turning velocity of the soft robot could be predicted by photographing the turning motion video. Due to the achievement of larger velocity by using square waveform in robot actuation, the input voltages in square waveform were applied on the prototype to describe the turning motion.

Through applying the asymmetrical signals on both actuators, motions of turning right and turning left were achieved successfully. Based on the trajectory of turning motion and corresponding time, the turning velocity was determined, and the maximum turning velocity was about 27 deg/s by using square waveform.

**Figure 16:**
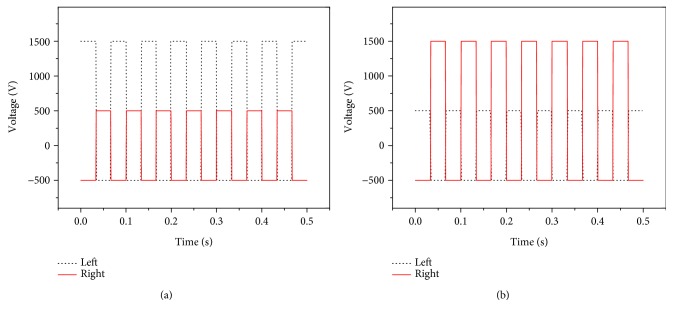
Input voltage signals in square waveform on both MFC actuators at 15 Hz in turning motion. (a) Turning right motion. (b) Turning left motion.

According to the control method of input voltage signals on both actuators, the turning motion was also achieved on the new soft robot prototype. To describe the turning motion of the new soft robot, the input voltage signals with 15 Hz in square waveform shown in Figure 16 were adopted, where the signals within 0.5 s were used as an example for description. In turning right motion, the input voltage on the right actuator was in the range of −500 V~+500 V, smaller than the voltage ranged from −500 V to +1500 V on the left actuator. For the turning left motion, the applied approach of input voltage signals on both actuators was contrary to that of turning right motion.

**Figure 17:**
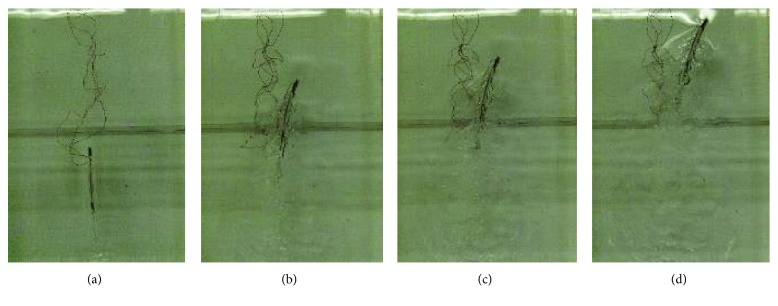
Turning right motion of new soft robot prototype at 15 Hz. (a) *T* = 0 s. (b) *T* = 0.3 s. (c) *T* = 0.4 s. (d) *T* = 0.6 s.

**Figure 18:**
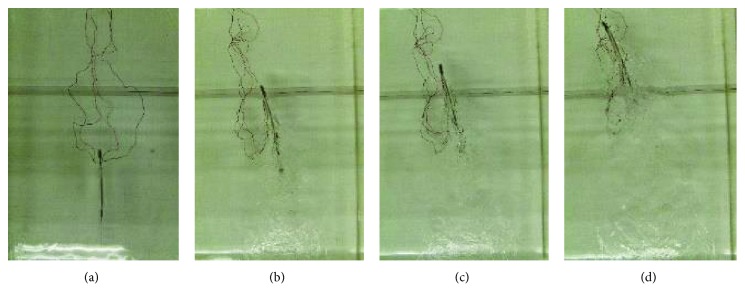
Turning left motion of new soft robot prototype at 15 Hz. (a) *T* = 0 s. (b) *T* = 0.3 s. (c) *T* = 0.4 s. (d) *T* = 0.6 s.

Based on these asymmetrical input voltage signals, the turning motions of the new soft robotic fish were achieved. Figures 17 and 18 present the turning right motion and turning left motion of the new soft robot prototype at its maximum swimming velocity by using a high-speed camera, respectively. Based on the trajectory of turning motion and corresponding time, the turning velocity was determined. The turning right motion with about 51.6 deg/s and turning left motion with about 53 deg/s were obtained at 15 Hz. A well-established experiment on a new soft robot prototype also validated the turning motion control.

## 6. Conclusions

In this paper, a new soft robotic fish using PFC was designed through the fluid-structural coupling analysis based on biomimetic approach. Through the experiment evaluation, it was confirmed that the better fish-like swimming performances were achieved successfully by the new soft robotic fish. The new soft robotic fish with high performance similar to those of real fishes had been developed successfully. In addition, the turning motion control of the soft robotic fish was established by controlling the input signals on both actuators. The turning left motion about 53 deg/s and turning right motion about 51.6 deg/s were achieved through the new soft robotic fish at the place where the maximum swimming velocity occurred.

## Figures and Tables

**Table 1:** Material properties of soft robotic fish.

Item	Weight	CFRP
Density (kg/m^3^)	7850	1643
Elastic modulus (GPa)	20	20.5
Poisson's ratio	0.3	0.3
Thickness (m)	0.0025	0.0002

**Table 2:** Range of key structural parameters of the soft robot.

Key structural parameters of soft robot	Range of optimization
A: caudal peduncle height (m)	0.001~0.064
B: caudal fin height (m)	0.001~0.064
C: body thickness (CFRP thickness) (m)	0.0001~0.001
D: radius of head weight (m)	0.001~0.007
Entire robot body length (m)	0.175

**Table 3:** Bending mode frequencies of the new soft robot in the fluid.

Item	Mode shape	Frequency (Hz)
1st mode	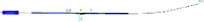	2.5
2nd mode	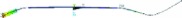	9.5
3rd mode	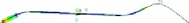	11.8

**Table 4:** Specifications of the new soft robotic fish.

Item	Specification
Caudal peduncle height (m)	0.003
Caudal fin height (m)	0.02
Radius of head weight (m)	0.007
Maximum body height (m)	0.064
Type of actuator	MFC 8528P1 × 2
Actuator dimensions (m)	0.103 × 0.035
Actuator active area (m)	0.085 × 0.028
Actuator for adhering	CFRP
Entire body length (m)	0.175
Adhesion bond	Epoxy 3M-DP460
Weight (kg)	0.0141

**Table 5:** Bending mode frequencies of the new soft robot prototype in the fluid.

Item	1st mode	2nd mode	3rd mode
Frequency (Hz)	2	10	12

**Table 6:** Comparison of swimming performance of the conventional soft robotic fish.

Soft robotic fish	Soft actuator	Maximum *S*_*w*_	Maximum speed (BL/s)
Kagawa University	ICPF	0.09	0.16
Polytechnic University of Madrid	SMA	0.2	0.1
Tokyo University	Electrostatic film	0.083	0.14
Michigan State University	IPMC	0.044	0.04848
This study	PFC	0.75	3.5

## Data Availability

The data used to support the findings of this study are available from the corresponding author upon request.
